# Comparative physiological and photosynthetic responses of three mangrove species to salinity stress and exogenous melatonin

**DOI:** 10.3389/fpls.2026.1966477

**Published:** 2026-10-13

**Authors:** Yun Wang, Kashif Ali Solangi, Xiqiang Song, Jinling Zhang, Farheen Solangi, Zexiu Yang

**Affiliations:** 1 Key Laboratory of Genetics and Germplasm Innovation of Tropical Special Forest Trees and Ornamental Plants (Ministry of Education), School of Tropical Agriculture and Forestry, Hainan University, Danzhou, China; 2 Hainan Institute of National Park, Haikou, China; 3 Research Centre of Fluid Machinery Engineering and Technology, Jiangsu University, Zhenjiang, China; 4 Hainan Evergreen Institute of Forestry Ecology Research, Haikou, China

**Keywords:** mangrove species, physiological parameters, exogenous melatonin, salt tolerance, chlorophyll fluorescence

## Abstract

Extreme salinity is a major environmental constraint affecting plant growth and physiological performance in coastal ecosystems. Although mangroves are naturally adapted to saline environments, their tolerance and physiological responses vary among species, and the effects of exogenous melatonin (MT) under different salinity levels remain insufficiently understood. This study compared the physiological and photosynthetic responses of *Aegiceras corniculatum*, *Kandelia obovata*, and *Rhizophora stylosa* under NaCl-only and corresponding NaCl + MT treatments. Seedlings were subjected for 84 days to seven treatments: control (T1), 100 mM NaCl (T2), 200 mM NaCl (T3), 400 mM NaCl (T4), 100 mM NaCl + 100 μM MT (T5), 200 mM NaCl + 100 μM MT (T6), and 400 mM NaCl + 100 μM MT (T7). Each species × treatment combination comprised one container–reservoir treatment experimental unit containing five seedling subsamples. At 200 mM NaCl, predicted maximum leaf area (K) was 14.6% and 43.0% higher in *A. corniculatum* and *K. obovata*, respectively, under the corresponding NaCl + MT treatment than under NaCl alone. Severe salinity (T4) net photosynthetic rate (P_N_) was 70.18% and 64.17% lower in *A. corniculatum* and *K. obovata*, respectively compared with the control. Whereas *R. stylosa* maintained comparatively higher photosynthetic activity across treatments. Furthermore, reductions were observed in other photosynthesis parameters, with *A. corniculatum* showing the greatest sensitivity, whereas *R. stylosa* maintained higher photosynthetic activity under saline conditions. Maximum PSII quantum efficiency (Fv/Fm) remained within a relatively narrow range across treatments, whereas photochemical and non-photochemical responses differed among species. Pigment responses were also species specific, at 200 mM NaCl, total chlorophyll in *K. obovata* was 58.0% higher under T6 than under the corresponding NaCl-only treatment (T3). These findings indicate that MT-associated responses varied with mangrove species and salinity level, with the clearest changes in specific growth, gas-exchange, photoprotective, and pigment traits under NaCl treatments. Because no MT-only treatment was included, the independent effect of MT under non-saline conditions could not be evaluated; moreover, the shared container–reservoir design limited independent treatment-level replication.

## 1 Introduction

Salinity is one of the major abiotic stresses limiting plant growth and physiological performance, particularly in coastal and intertidal ecosystems (F. Wang et al., 2024). Mangroves are halophytic plants that naturally inhabit saline coastal zones and can tolerate multiple environmental constraints, including high salinity, tidal inundation, waterlogging, oxygen-deficient sediments, drought, high temperature, and fluctuating salt concentrations (Ramachandran et al., 2025). However, among these stress factors, excessive salinity remains one of the most significant constraints on mangrove seedling establishment and early growth (W. Wang et al., 2024). High salinity induces osmotic stress, promotes the accumulation of toxic Na^+^ and Cl^−^ ions, disturbs nutrient uptake and ion homeostasis, and enhances the production of reactive oxygen species (ROS) (Li et al., 2025). These physiological disturbances can damage cell membranes and photosynthetic structures, reduce stomatal conductance and carbon assimilation, and ultimately suppress leaf expansion and overall plant development (Solangi et al., 2022). Although mangroves have specialized adaptive mechanisms such as salt exclusion, salt secretion, ion compartmentalization, and osmotic adjustment, their tolerance capacity is not unlimited (Sudhir et al., 2022). Severe salt stress can still impair photosynthetic efficiency and weaken seedling survival, especially during nursery cultivation and restoration in highly saline coastal environments (Balasubramaniam et al., 2023).

The application of exogenous plant growth regulators has been widely investigated as a potential approach to improve plant tolerance to salinity and other abiotic stresses (Quamruzzaman et al., 2021). Among these regulators, melatonin (N-acetyl-5-methoxytryptamine) has received increasing attention because of its diverse roles in plant growth, development, and stress defense (Altaf et al., 2022). Melatonin (MT) is a naturally occurring, low-molecular-weight indoleamine that participates in several physiological processes in plants (Altaf et al., 2021). Previous studies have reported that MT can enhance plant tolerance to salinity, drought, heat, cold, heavy-metal toxicity, and chemical stress, while also contributing to defense responses against pathogens (Altaf et al., 2022). In addition, MT has been shown to promote seed germination, root development, seedling growth, photosynthetic performance, antioxidant activity, and fruit maturation, while delaying leaf senescence (Liang et al., 2026). Previous studies have associated MT with the regulation of redox homeostasis and antioxidant defense responses under abiotic stress (Zeng et al., 2022). In other plant systems exogenous MT has been reported to reduce O_2_•^−^ and H_2_O_2_ accumulation while increasing peroxidase and catalase activities (Nasser et al., 2022). These literature-reported responses provide a basis for investigating MT-associated physiological changes in mangrove performance under salinity stress. These mechanisms were not directly measured in the present study.

Recent evidence has highlighted the potential role of MT in regulating stress responses in mangroves. For example, Li et al. (2026) reported that exogenous MT reduced cold-induced leaf injury, and oxidative stress, and limited PSI/PSII photoinhibition in *Kandelia obovata* (*K. obovata*). Similarly, another study showed that endogenous serotonin- and melatonin-related redox regulation improved ROS/RNS homeostasis and reduced PSI/PSII photoinhibition under cold stress (Li et al., 2024). Furthermore, improved stress tolerance in mangroves was associated with reduced leaf damage, maintenance of photosynthetic capacity, and regulation of genes related to melatonin and ABA biosynthesis (Shan et al., 2025). Together, these studies indicate that melatonin-related pathways are associated with physiological and photosynthetic responses to stress in mangroves. However, previous studies have focused primarily on cold stress whereas comparative evidence for exogenous MT-mediated responses to salinity in mangrove species remains limited. It remains unclear whether MT-associated changes in leaf growth, gas exchange, chlorophyll fluorescence, and photosynthetic pigments differ among mangrove species and salinity levels. Therefore, the present study compared *Aegiceras corniculatum* (*A*. *corniculatum*), *K*. *obovata*, and *Rhizophora stylosa* (*R*. *stylosa*), using NaCl-only and corresponding NaCl + MT treatments. These species exhibit contrasting growth and regenerative characteristics, providing a suitable basis for species-level comparison (Zhang et al., 2021).

The novelty of this study lies in the comparative evaluation of physiological and photosynthetic responses of three mangrove species across NaCl-only and corresponding NaCl + MT treatments. Using measurements of leaf growth, photosynthetic light response, gas exchange, chlorophyll fluorescence, and photosynthetic pigments, this study aimed to (i) determine species-specific responses to increasing salinity and (ii) evaluate whether 100 μM MT modifies these responses at corresponding salinity levels. We hypothesized that salinity would reduce growth and photosynthetic performance in a species-dependent manner and that MT-associated responses would vary with both species and salinity level.

## 2 Materials and methods

### 2.1 Plant materials and seedling acclimation

The experiment was conducted in a greenhouse at Hainan University, Hainan Island, China. During the experiment, greenhouse conditions were maintained at approximately 28/23 °C (day/night), with a relative humidity of approximately 82%. One-year-old seedlings of three mangrove species, *A*. *corniculatum*, *K*. *obovata*, and *R*. *stylosa*, were collected from the Dongzhai Harbor National Mangrove Nature Reserve in northeastern Hainan Island, China as shown in **Figure 1**. Healthy seedlings approximately 60–70 cm in height were selected to minimize variation in their initial growth conditions. The seedlings were transported to the greenhouse along with soil collected from the same area. During collection and transportation, each seedling retained its original root-zone soil within an individual plastic nursery bag, thereby minimizing disturbance to the root system. After arrival, excess mud and surface debris were gently removed using tap water, while care was taken to avoid damage to the root systems. The seedlings were subsequently placed in experimental containers containing the collected mangrove soil. The experimental containers measured approximately 93 × 60 × 25 cm (length × width × height). The collected mangrove soil had an initial pH of 6.1–6.5 and electrical conductivity (EC) of 2.61–2.99 dS m^−1^.

**Figure 1:**
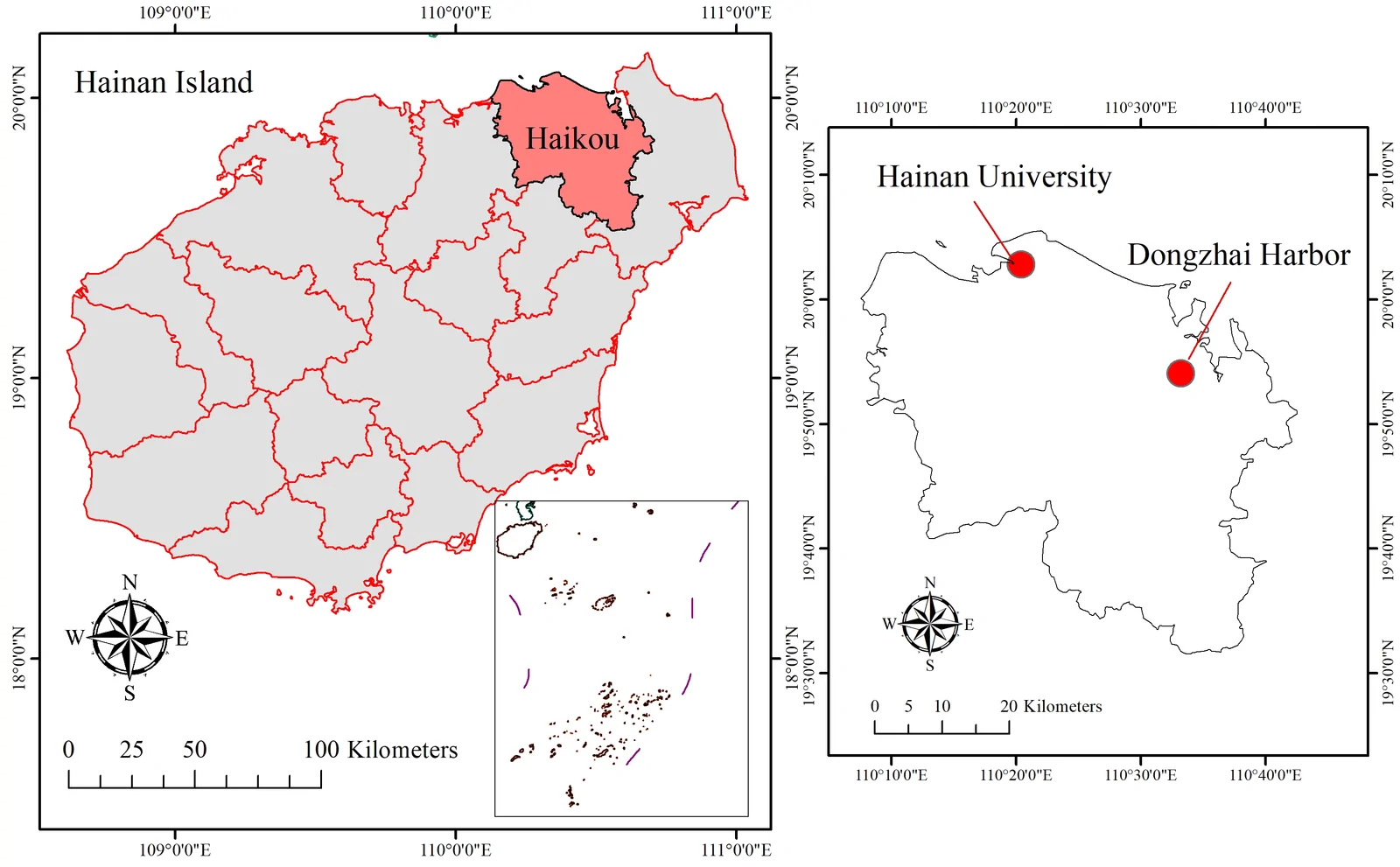
Location of Hainan University, where the greenhouse experiment was conducted, and Dongzhai Harbor, where the mangrove seedlings were collected, Hainan Island.

Before the treatments were initiated, all seedlings were acclimated to greenhouse conditions for three days. During the acclimation period, the seedlings received Hoagland nutrient solution diluted with tap water to provide essential nutrients and reduce stress from transportation and transplantation. The three-day acclimation period was used because the root-zone soil remained intact during transportation and placement in the experimental containers, thereby minimizing transplantation disturbance, and no visible symptoms of transplantation injury were observed before treatment initiation.

### 2.2 Experimental design and salt–melatonin treatments

The experiment included seven treatment combinations involving three mangrove species and different NaCl and melatonin (MT) conditions. The treatments comprised a non-saline control without MT (T1), three only NaCl treatments (T2: 100 mM NaCl; T3: 200 mM NaCl; and T4: 400 mM NaCl), and three corresponding NaCl treatments supplemented with 100 μM MT (T5: 100 mM NaCl + 100 μM MT; T6: 200 mM NaCl + 100 μM MT; and T7: 400 mM NaCl + 100 μM MT). Melatonin was applied at a constant concentration of 100 μM under T5, T6 and T7 to evaluate treatment-specific physiological and photosynthetic responses to MT supplementation under salinity stress. Because an MT-only treatment at 0 mM NaCl was not included, the experiment did not constitute a complete NaCl × MT factorial design. MT-associated responses were therefore evaluated only by comparing each NaCl + MT treatment with its corresponding NaCl-only treatment at the same salinity level. Independent MT main effects and a full NaCl × MT interaction were not estimated.

A total of 21 experimental container–reservoir units were established, corresponding to three mangrove species × seven treatments. Each experimental container was connected to its own independent 34-L treatment reservoir and contained five individual seedlings. Because the five seedlings within each species × treatment combination shared the same container and treatment solution, the container–reservoir pair constituted the experimental treatment unit, while the five seedlings were treated as subsamples rather than independent treatment replicates. Melatonin (purity >99%; molecular weight 232.28 g mol^−1^) was prepared at a nominal final concentration of 100 μM. For each 34-L MT treatment solution, 0.790 g MT was first dissolved in 10 mL ethanol and subsequently diluted into the corresponding treatment solution. The non-MT treatments (Control (T1)–T4) did not receive ethanol. For T5–T7, MT was mixed directly with the corresponding NaCl treatment solution and supplied through the same automated irrigation system.

The NaCl and MT treatment solutions were supplied through a fully automated irrigation system connected to the experimental containers as shown in **Figure 2**. The irrigation system was operated for 8 h per day, during which the respective treatment solutions were delivered to the mangrove root zone, flooding the root zone to approximately 2–3 inches above the root-zone level. During the irrigation period, the treatment solution was recirculated between each experimental container and its corresponding reservoir. After the 8-hours irrigation period, the solution was returned to the same reservoir. No additional treatment solution was supplied during the remaining 16 h.

**Figure 2:**
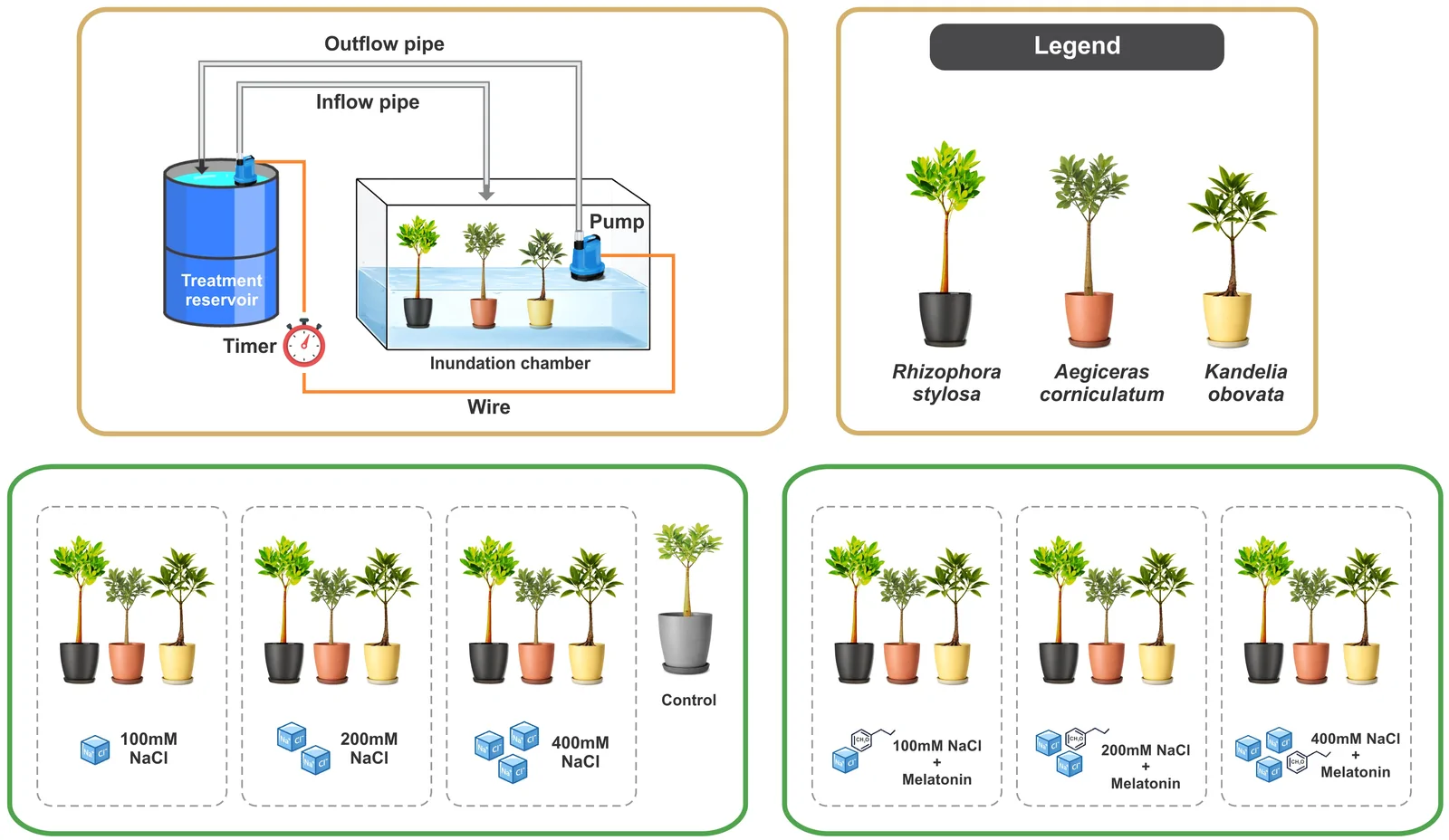
Schematic representation of the automated tidal-irrigation system and NaCl–melatonin treatment design for three mangrove species. One-year-old seedlings of *Aegiceras corniculatum*, *Kandelia obovata*, and *Rhizophora stylosa* were exposed for 84 days to seven treatments: control (T1), 100, 200, and 400 mM NaCl, and the same NaCl concentrations combined with 100 μM melatonin. Each species × treatment combination consisted of one container–reservoir experimental unit containing five seedling subsamples. Treatment solutions were supplied to the root zone using an automated irrigation system for 8 h day^−1^.

The treatment solutions were completely replaced every 30 days, while the same NaCl and MT concentrations were maintained throughout the experiment. This daily watering regime was designed to create a controlled tidal-type irrigation cycle suitable for mangrove seedlings. Each species × treatment combination contained five seedling subsamples within one container reservoir treatment unit. A total of 105 seedlings were used across the 21 treatment units (three species × seven treatments × five seedlings). All treatments were maintained for 84 days. To reduce greenhouse positional effects, the experimental containers were repositioned every 15 days, resulting in six positional changes during the experiment. In addition, the overall arrangement of species and containers was changed during each 30-day solution renewal. The overall experimental arrangement and treatment design are presented in **Figure 2**.

### 2.3 Logistic modeling of leaf growth dynamics

Leaf growth dynamics of three mangrove species were evaluated under different NaCl concentrations, with and without MT treatments using a four-parameter logistic growth model. This model is commonly used to describe sigmoidal biological growth patterns over time (Archontoulis and Miguez, 2015). The same selected leaf on each seedling was repeatedly measured throughout the experiment. Leaf length and maximum leaf width were measured using a measuring scale, and estimated leaf area was calculated as the product of leaf length and maximum width (L × W). Leaf growth was measured over time with assessments repeated every 2 days, continuing until 84 days. For each species × treatment combination, one selected leaf from each of the five seedlings was included in the final growth dataset, resulting in five leaves per treatment unit. At each measurement date, the five leaf-area measurements were averaged to obtain the treatment-level mean trajectory. The fitted treatment trajectories were compared descriptively rather than through inferential statistical tests, and no inferential comparisons across time were performed.

The logistic model was fitted using Equation 1:

(1) $$Y=y_{0}+\frac{K-y_{0}}{1+e^{-r\left(t-t_{0}\right)}}$$

where *Y* represents the predicted estimated leaf area at time *t*, *t* represents days after treatment, *y*_0_ represents the lower asymptotic leaf area parameter, *K* represents the predicted maximum estimated leaf area, *r* represents the relative growth rate, and *t*_0_ represents the inflection point, corresponding to the time of maximum growth rate.

For each species × treatment combination, the four-parameter logistic model was fitted separately to the treatment-level mean leaf-area trajectory using nonlinear regression for descriptive characterization. The estimated model parameters included *y*_0_, *K*, r, and t_0_. Because the biological interpretation focused on growth potential, growth rate, and the timing of the fastest growth phase, *K*, *r*, and *t*_0_ are reported in **Table 1**. Parameter estimates are presented as estimate ± standard error (SE), together with their 95% confidence intervals (CI). *K* was used to represent asymptotic leaf-area potential, *r* to describe the relative growth rate, and *t*_0_ to identify the modeled inflection time. Model performance was evaluated using the coefficient of determination (R^2^) and root mean square error (RMSE). Higher R^2^ values and lower RMSE values indicate better model fitting.

**Table 1:** Selected parameter estimates from the four-parameter logistic growth model for leaf-area development under NaCl and melatonin treatments.

Species	Treatment	K (cm^2^) estimate ± SE (95% CI)	r (day^−1^) estimate ± SE (95% CI)	t_0_ (days) estimate ± SE (95% CI)	R^2^	RMSE
*A. corniculatum*	Control	53.70 ± 0.42 (52.84–54.57)	0.186 ± 0.022 (0.142–0.231)	11.23 ± 1.11 (8.94–13.52)	0.990	1.491
	100 mM	15.73 ± 0.30 (15.12–16.35)	0.091 ± 0.023 (0.044–0.139)	10.50 ± 7.08 (−4.15–25.15)	0.963	0.709
	200 mM	28.39 ± 0.31 (27.75–29.02)	0.268 ± 0.035 (0.197–0.340)	23.66 ± 0.56 (22.51–24.81)	0.986	1.053
	400 mM	14.10 ± 0.20 (13.68–14.52)	0.103 ± 0.020 (0.061–0.145)	10.78 ± 4.72 (1.01–20.55)	0.975	0.536
	100 mM + MT	14.80 ± 0.15 (14.49–15.10)	0.134 ± 0.025 (0.083–0.185)	7.52 ± 3.73 (−0.20–15.23)	0.978	0.472
	200 mM + MT	32.54 ± 0.35 (31.82–33.25)	0.186 ± 0.026 (0.132–0.240)	12.75 ± 1.25 (10.17–15.33)	0.985	1.218
	400 mM + MT	15.32 ± 0.34 (14.63–16.02)	0.085 ± 0.022 (0.039–0.130)	12.27 ± 7.27 (−2.76–27.31)	0.962	0.727
*K. obovata*	Control	43.22 ± 0.80 (41.56–44.87)	0.054 ± 0.009 (0.035–0.073)	10.85 ± 7.43 (−4.51–26.22)	0.991	0.803
	100 mM	21.26 ± 0.31 (20.61–21.91)	0.085 ± 0.011 (0.062–0.107)	20.03 ± 2.46 (14.95–25.11)	0.989	0.610
	200 mM	25.84 ± 0.67 (24.45–27.24)	0.078 ± 0.017 (0.042–0.114)	18.71 ± 4.98 (8.41–29.00)	0.971	1.196
	400 mM	18.25 ± 0.16 (17.92–18.58)	0.070 ± 0.013 (0.043–0.096)	−1.43 ± 11.69 (−25.61–22.76)	0.986	0.298
	100 mM + MT	27.08 ± 0.20 (26.67–27.49)	0.151 ± 0.007 (0.136–0.165)	33.55 ± 0.33 (32.87–34.24)	0.998	0.496
	200 mM + MT	36.96 ± 1.18 (34.52–39.40)	0.069 ± 0.009 (0.051–0.086)	38.84 ± 1.42 (35.90–41.79)	0.990	1.024
	400 mM + MT	19.74 ± 0.28 (19.17–20.31)	0.062 ± 0.007 (0.048–0.075)	17.38 ± 3.26 (10.64–24.12)	0.995	0.329
*R. stylosa*	Control	36.88 ± 0.67 (35.50–38.27)	0.091 ± 0.008 (0.074–0.108)	35.03 ± 0.91 (33.15–36.92)	0.993	1.043
	100 mM	24.55 ± 0.42 (23.69–25.42)	0.070 ± 0.011 (0.047–0.094)	14.98 ± 4.82 (5.02–24.94)	0.987	0.666
	200 mM	45.26 ± 1.21 (42.75–47.77)	0.100 ± 0.022 (0.055–0.146)	21.59 ± 3.18 (15.01–28.18)	0.965	2.786
	400 mM	47.00 ± 1.87 (43.13–50.87)	0.066 ± 0.006 (0.053–0.079)	51.89 ± 1.50 (48.78–54.99)	0.994	0.909
	100 mM + MT	15.63 ± 0.47 (14.67–16.60)	0.054 ± 0.019 (0.015–0.094)	4.86 ± 19.61 (−35.72–45.43)	0.964	0.510
	200 mM + MT	39.74 ± 0.32 (39.09–40.40)	0.101 ± 0.010 (0.080–0.123)	11.54 ± 2.46 (6.45–16.62)	0.993	0.824
	400 mM + MT	32.37 ± 0.88 (30.55–34.19)	0.062 ± 0.008 (0.045–0.079)	28.50 ± 2.33 (23.69–33.31)	0.992	0.828

Values are presented as parameter estimate ± SE, with 95% confidence intervals in parentheses. *K*, predicted maximum leaf area; *r*, relative growth-rate parameter; *t*_0_, inflection time; SE, standard error; CI, confidence interval; R^2^, coefficient of determination; RMSE, root mean square error; MT, melatonin.

The percentage change in the predicted maximum estimated leaf area under each treatment relative to the control was calculated as Equation 2:

(2) $$\text{Change relative to T}1=\frac{K_{\text{treatment}}-K_{\text{T}1}}{K_{\text{T}1}}\times 100$$

where *K*_treatment_ represents the predicted maximum estimated leaf area under a given treatment and *K*_T1_ represents the corresponding value under the control treatment.

The relative effect of melatonin at each salinity level was calculated by comparing the NaCl treatment supplemented with melatonin with its corresponding NaCl-only treatment using Equation 3:

(3) $$\text{Melatonin effect}=\frac{K_{\text{NaCl}+\text{MT}}-K_{\text{NaCl}}}{K_{\text{NaCl}}}\times 100$$

where $K_{\text{NaCl+MT}}$ represents the predicted maximum estimated leaf area under the combined NaCl and melatonin treatment, and *K*_NaCl_ represents the predicted maximum estimated leaf area under the corresponding NaCl-only treatment. Positive percentage values indicated higher K under NaCl + MT than under the corresponding NaCl-only treatment, whereas negative values indicated lower K. The fitted parameters were subsequently used to compare species-specific growth patterns and responses to salinity and melatonin treatments descriptively.

### 2.4 Photosynthetic light-response curves

Photosynthetic light-response curves were measured using an LI-6800 portable photosynthesis system equipped with a 6800-01A leaf chamber (LI-COR Biosciences, Lincoln, NE, USA). Measurements were performed on the third or fourth healthy, fully expanded mature leaf. Measurements were obtained from five seedling subsamples within each species × treatment combination. After each selected leaf was enclosed in the chamber, sufficient time was allowed for the gas-exchange parameters to stabilize. Photosynthetic photon flux density (PPFD) was subsequently decreased stepwise using the following sequence: 1800, 1500, 1200, 900, 600, 300, 200, and 100 (μmol m^−2^ s^−1^). At each PPFD level, data were recorded after the measured parameters had stabilized and were averaged over a 15-s recording period. During measurement, the reference CO_2_ concentration was maintained at approximately 400 μmol mol^−1^, the gas-flow rate at approximately 500 μmol s^−1^, and the chamber heat-exchanger temperature at 30 °C. The water-vapour concentration of the reference air was maintained at approximately 37.94 mmol mol^−1^. Photosynthetic light-response curves were generated by plotting the mean net photosynthetic rate (P_N_) against PPFD for each species and treatment. The resulting curves were used to compare the photosynthetic performance and light-use responses of the three mangrove species under NaCl and combined NaCl–melatonin treatments.

### 2.5 Leaf gas-exchange measurements

Leaf gas-exchange parameters were measured using the LI-6800 portable photosynthesis system described in section 2.4. Each leaf was allowed to acclimate inside the chamber until stable gas-exchange readings were obtained. All measurements were performed under uniform chamber conditions to ensure comparability among species and treatments. Measurements were performed between 8:30 am to 11: 30 a.m. The reference CO_2_ concentration was maintained at approximately 400 μmol mol^−1^, the gas-flow rate at approximately 500 μmol s^−1^, and the chamber heat-exchanger temperature at 30 °C. The reference water-vapour concentration was maintained at approximately 37.94 mmol mol^−1^. The recorded parameters included net photosynthetic rate (P_N_), stomatal conductance (G_S_), intercellular CO_2_ concentration (Ci), and transpiration rate (Tr).

### 2.6 Determination of chlorophyll fluorescence

Chlorophyll fluorescence parameters were measured using a PAM-2500 portable chlorophyll fluorometer (Walz, Germany). The measured parameters included minimum fluorescence (F_0_), maximum fluorescence Fm, variable fluorescence Fv, maximum quantum efficiency of photosystem II Fv/Fm, effective quantum yield of photosystem II (ΦPSII), and electron transport rate (ETR). Variable fluorescence was calculated as Fv = Fm − F_0_. The Fv/Fm measurements were conducted after leaves had been dark-adapted for 20 min, whereas ΦPSII was measured under natural light conditions between 10:00 and 11:00. Non-photochemical quenching was calculated as $\text{NPQ}={\text{F}}_{\text{m}}/{\text{F}}_{\text{m}}^{\prime }-1$, where ${\text{F}}_{\text{m}}^{\prime }$ represents the maximum fluorescence of the light-adapted leaf induced by a saturating pulse. The electron transport rate was calculated using Equation 4.

(4) ETR=ΦPSII×PPFD×0.5×0.84

where PPFD represents the photosynthetic photon flux density, 0.5 represents the assumed fraction of absorbed light distributed to photosystem II, and 0.84 represents the assumed leaf-light absorptance (Chen et al., 2023). One leaf was measured per seedling, and the measurements from five seedlings per species–treatment combination were used for subsequent data summarization.

### 2.7 Determination of photosynthetic pigment content

The contents of chlorophyll a (Chl–a), chlorophyll b (Chl–b), carotenoids (Car) and total chlorophyll (Chl–a + Chl–b), were determined using an ethanol–acetone extraction method. For each species–treatment combination, leaf samples were collected from five seedlings. Samples from the five seedlings were processed separately. Leaf tissue was collected from the leaves previously used for photosynthetic measurements. Fresh leaf tissue (0.5 g) was cut into small pieces and immersed in 10 mL of an ethanol–acetone mixture (1:1, v/v). The samples were incubated in darkness for 24 h at room temperature until the leaf tissue was completely decolorized. The absorbance of the pigment extract was measured at 663, 645, and 470 nm using a UV–visible spectrophotometer (UV-5000PC, Shanghai Yuanxi Instrument Co., Ltd., Shanghai, China).

The concentrations of chlorophyll a, chlorophyll b, and total chlorophyll in the extract were calculated using Equations 5–7, respectively (Lichtenthaler and Babani, 2022).

(5) $$\text{Chl–a}=12.72{\text{A}}_{663}-2.59{\text{A}}_{645}$$

(6) $$\text{Chl–b}=22.88{\text{A}}_{645}-4.67{\text{A}}_{663}$$

(7) $$\text{Chl–T}=8.05{\text{A}}_{663}+20.29{\text{A}}_{645}$$

The carotenoid concentration was calculated using Equation 8.

(8) $${\text{C}}_{\text{Car}}={(1000}_{\text{A}470}-3.27_{\text{Chl-a}}-104_{\text{Chl-b}})/229$$

where A_663_, A_645_ and A_470_ represent the absorbance values measured at 663, 645, and 470 nm, respectively, and C_Chl-a_, C_Chl-b_,C_Total_ Chl, and C_Car_ represent the pigment concentrations in the extract.

The pigment content on a fresh-weight basis was calculated using Equation 9.

(9) $$\text{Pigment content}\left({\text{mg g}}^{-1}\text{ FW}\right)=\frac{C\times V}{1000m}$$

where *C* is the pigment concentration in the extract (mg L^−1^), *V* is the total extract volume (10 mL), and *m* is the fresh weight of the leaf sample (0.5 g).

### 2.8 Statistical analysis

Each species × treatment combination was represented by one container–reservoir experimental unit containing five seedling subsamples. Because the five seedlings within each unit shared the same treatment solution, treatment responses were summarized descriptively rather than using treatment-level inferential comparisons. Gas-exchange, chlorophyll fluorescence, and pigment responses were summarized separately for each species using mean ± SD and percentage changes. Leaf-growth trajectories were characterized using the four-parameter logistic model described in Section 2.3, with fitted parameters interpreted descriptively. Graphs were prepared by using Origin Pro (Northampton, MA, USA).

## 3 Results

### 3.1 Leaf area growth model response under NaCl stress and melatonin application

The four-parameter logistic model showed clear species- and treatment-specific patterns in predicted asymptotic leaf area (K) (**Table 1**). In *A. corniculatum*, K was 14.6% higher under 200 mM NaCl + MT than 200 mM NaCl. In *K. obovata*, K was 27.4%, 43.0%, and 8.2% higher under the corresponding NaCl + MT treatments at 100, 200, and 400 mM NaCl, respectively. In *R. stylosa*, K was 36.3%, 12.2%, and 31.1% lower under NaCl + MT than under the corresponding NaCl-only treatments at 100, 200, and 400 mM NaCl, respectively. The fitted models showed strong agreement with the observed leaf-area trajectories.

### 3.2 Photosynthetic light-response curves

The photosynthetic light-response curves (PLC) showed clear difference among species and treatment- under salinity and MT application, as shown in **Figure 3**. Usually salinity reduced the light-response capacity, especially under 400 mM, while MT partially improved PLC values under saline conditions, particularly in T6 (200 mM NaCl + MT). *A. corniculatum* and *K. obovata* generally showed reduced photosynthetic rates under salinity treatments, with higher rates under control conditions, whereas *A. corniculatum* species showed higher PLC values in the T6 (200 mM NaCl + MT), and *K. obovata* presented better PLC values in T3–200 mM NaCl in comparison with other treatments. However, in *R. stylosa* species, PLC values increased in the T2 (100 mM NaCl) and T6 (200 mM NaCl + MT) as compared to control. Overall, the light-response curve suggested that salinity reduced photosynthetic capacity in *A. corniculatum* and *K. obovata*, while *R. stylosa* showed better performance and tolerance under stress.

**Figure 3:**
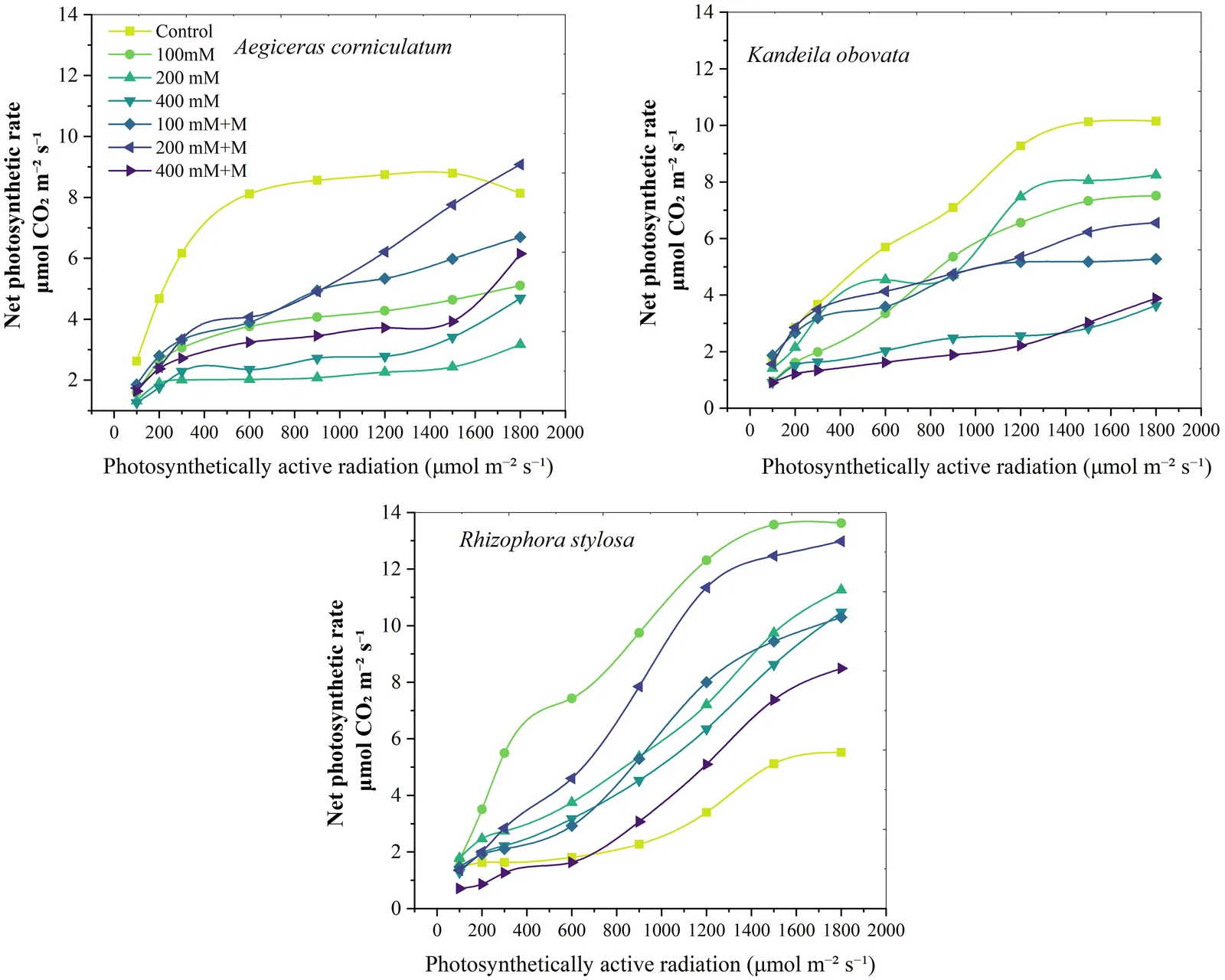
Photosynthetic light-response curves of three different mangrove species under salinity and melatonin treatments. T1 = control; T2 = 100 mM NaCl; T3 = 200 mM NaCl; T4 = 400 mM NaCl; T5 = 100 mM NaCl + 100 μM MT; T6 = 200 mM NaCl + 100 μM MT; and T7 = 400 mM NaCl + 100 μM MT. Curves are based on mean measurements from five seedling subsamples within each species × treatment unit.

### 3.3 Response of photosynthesis parameters

The net photosynthetic (P_N_) showed species and treatment dependent responses in **Figure 4**. Among the three species, *R. stylosa* consistently maintained the highest P_N_ values across all treatments, whereas *A. corniculatum* and *K. obovata* exhibited the greatest reduction of 70.18% and 64.17%, respectively, under T4 (400 mM NaCl) compared with the control. MT-associated changes in P_N_ were evaluated against the corresponding NaCl-only treatment (T5 vs T2, T6 vs T3, and T7 vs T4) and differed among species and salinity levels. In *R. stylosa*, P_N_ increased by 6.70% under 100 mM NaCl and by 5.11% under 200 mM NaCl + MT, compared with the control.

**Figure 4:**
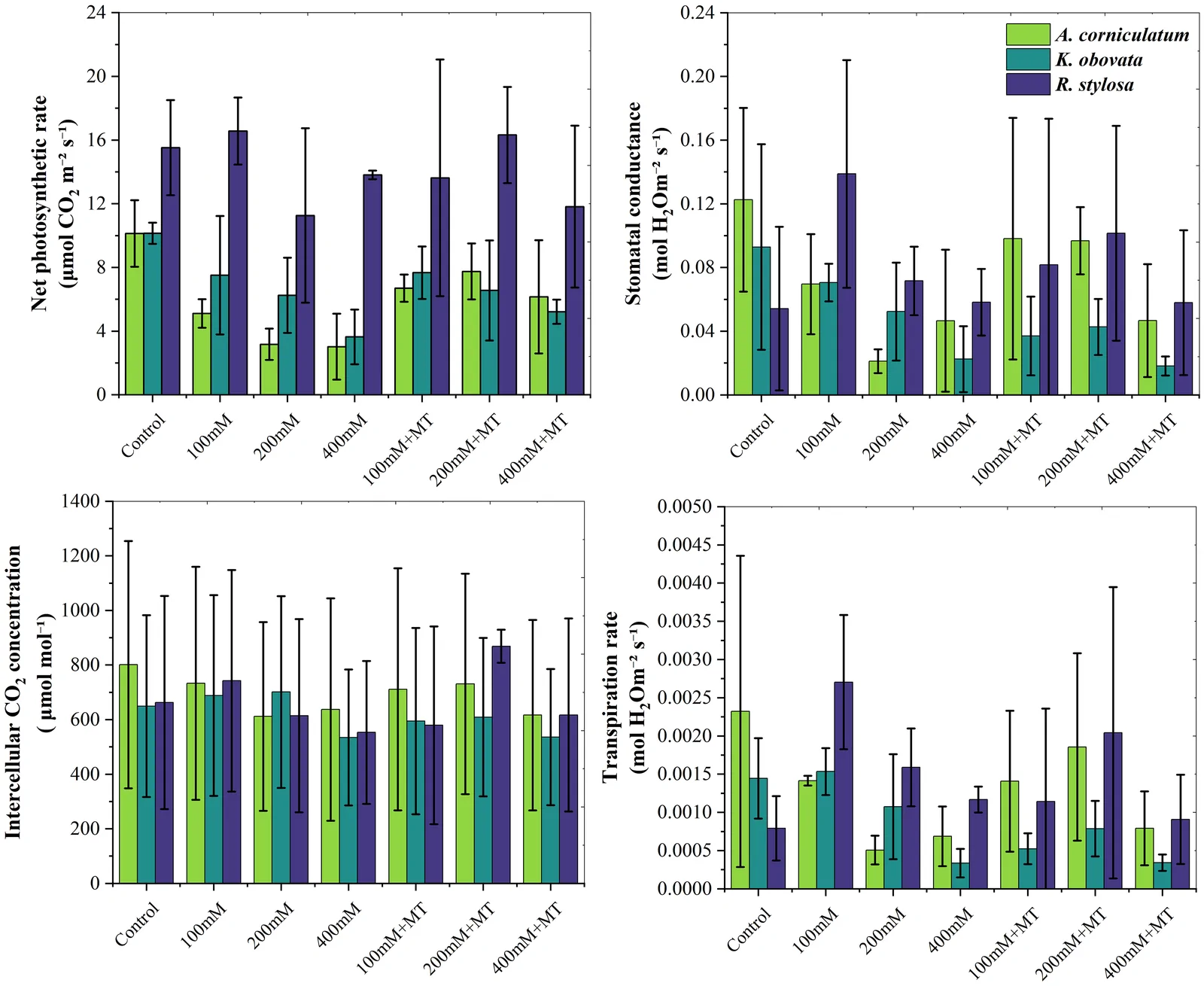
Effects of NaCl stress and melatonin application on photosynthetic gas-exchange parameters including; net photosynthetic, stomatal conductance intercellular CO_2_ concentration and transpiration rate in three mangrove species. Note: Values are presented as mean ± SD of five seedling subsamples within each species × treatment unit. Error bars represent variability among seedling subsamples and do not represent independent treatment-level replication.

Furthermore, stomatal conductance (G_S_) values showed clear species-specific responses to NaCl and MT treatments. In *A. corniculatum*, G_S_ decreased by 43.26%, 82.60%, and 61.99% under 100 mM, 200 mM, and 400 mM NaCl treatments compared to the control. MT applications increased G_S_ compared with the corresponding only NaCl treatments. In *K. obovata*, G_S_ values decreased by 23.95%, 43.62%, and 75.79% under 100, 200, and 400 mM NaCl, respectively, compared with the control. Under MT application, *K. obovata* also exhibited a pronounced reduction in G_S_ values. In contrast, *R. stylosa* exhibited higher G_S_ under several treatments, with the greatest increase (156.18%) observed at 100 mM NaCl relative to the control. Overall, G_S_ responses were more strongly reduced by NaCl in *A. corniculatum* and *K. obovata* than in *R. stylosa*. Furthermore, NaCl and MT application had only a minor influence on Intercellular CO_2_ concentration (Ci). The Ci values suggested that internal CO_2_ concentration was comparatively stable despite changes in photosynthesis. Compared with control, T4 and T7 (400 mM NaCl and 400 mM NaCl + MT) reduced the Ci by 23.6% and 23.1%, respectively, in *A. corniculatum*. In *K. obovata*, the maximum reduction in Ci was observed under T4 (400 mM NaCl), decreasing by 17.65%, while T7 (400 mM NaCl + MT) observed almost similar reduction of 17.43% with control. In *R. stylosa*, the greatest decline in Ci occurred under T4 (400 mM NaCl), with a 16.54% reduction relative to the control, whereas the largest decrease under MT treatment was recorded under T5(100 mM + MT), declining by 12.61% relative to the control. Additionally, results of transpiration rate (Tr) also differed among the three mangrove species under salinity and MT treatments. Overall, Tr declined under increasing NaCl, particularly in *A. corniculatum* and *K. obovata*. In *A. corniculatum*, Tr decreased by 78.18% under T3 (200 mM NaCl), while under T6 (200 mM NaCl + MT), the reduction was 20.53% compared with the control. In *K. obovata*, Tr increased slightly by 6.22% under 100 mM NaCl relative to the control. But declined under 200 mM and 400 mM by 25.72% and 76.84%, respectively. In the MT treatments, *K. obovata* also showed reduced Tr values. In contrast, *R. stylosa* showed higher Tr under most treatments, especially under T6 (200 mM NaCl+ MT 157.95%) and T3 (200 mM NaCl; 100.83%) relative to the control. Overall, gas-exchange responses differed among species and salinity levels, and the magnitude of MT-associated changes depended on the corresponding NaCl treatment.

### 3.4 Response of chlorophyll fluorescence parameters

Chlorophyll fluorescence responses varied among species and treatments shown in **Figure 5**. The maximum quantum yield of photosystem II (Fv/Fm) showed only slight variation among species and treatments. Across treatments, Fv/Fm remained within a relatively narrow range (approximately 0.81–0.86) across all three species, indicating limited variation in maximum PSII photochemical efficiency. Treatment-related changes were more apparent in ΦPSII and NPQ. ΦPSII varied moderately among treatments, whereas NPQ showed stronger treatment-related variation, particularly in *A. corniculatum* and *K. obovata*. Overall, the fluorescence responses indicate species-specific differences in photochemical adjustment and non-photochemical energy dissipation under NaCl and NaCl + MT treatments.

**Figure 5:**
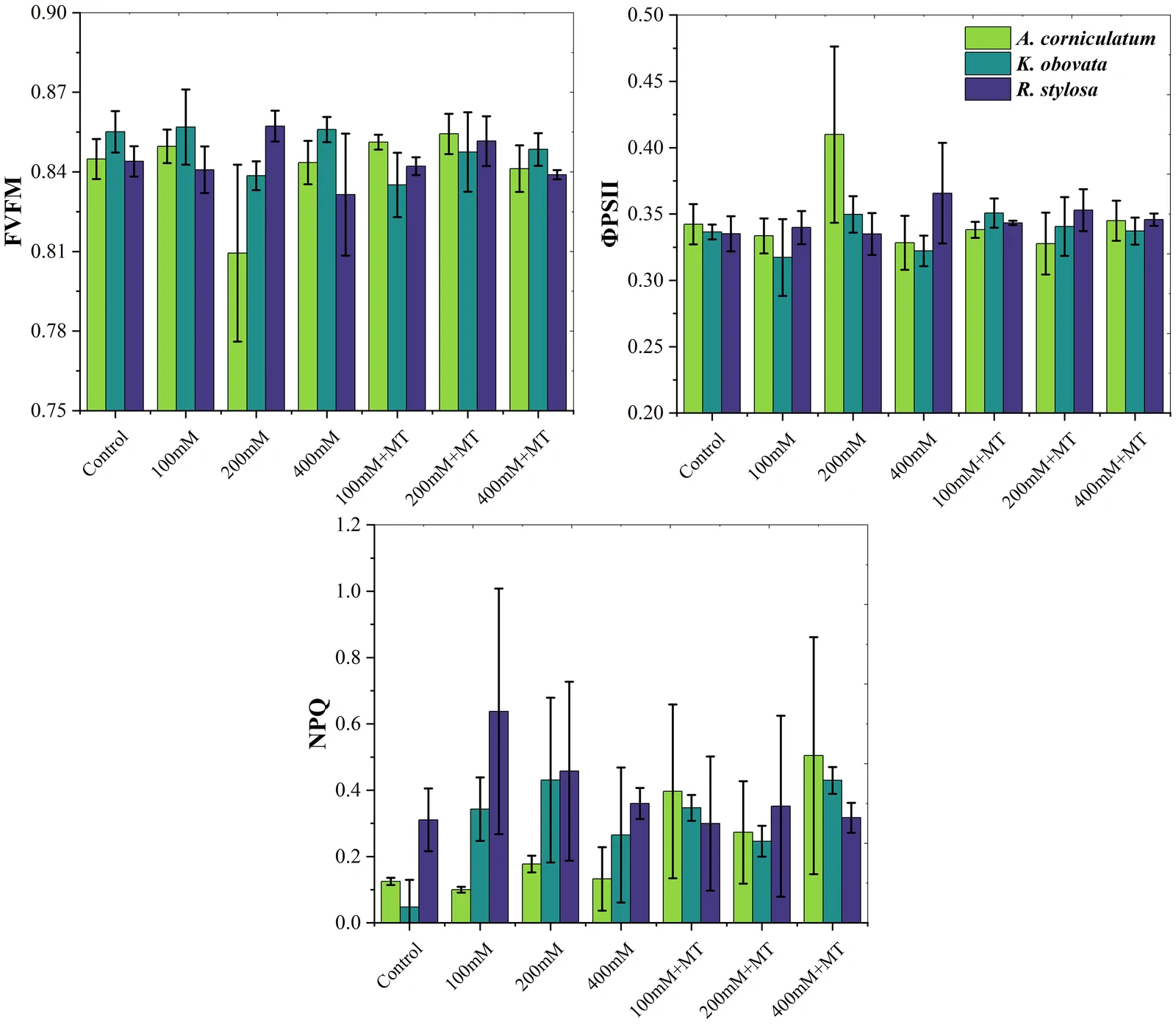
Effects of NaCl stress and melatonin application on chlorophyll fluorescence parameters including maximum quantum yield of photosystem II (Fv/Fm), effective quantum yield of photosystem II (ΦPSII), and non-photochemical quenching (NPQ) of three mangrove species. Values are presented as mean ± SD of five seedling subsamples within each species × treatment unit. Error bars represent variability among seedling subsamples and do not represent independent treatment-level replication.

### 3.5 Response of photosynthetic pigments

The results of photosynthetic pigment content, including chlorophyll a (Chl–a), chlorophyll b (Chl–b), carotenoids (Car), and total chlorophyll (Chl–T), differed among treatments and species as presented in **Figure 6**. *R. stylosa* showed more NaCl stability with MT treatment. In *A. corniculatum* Chl–a increased by T5 by 21.52% compared to the control. While Chl–b was 31.28% higher under T4 in *A. corniculatum* species compared to control. *In K. obovata*, Chl–b was 4% higher under T6 (200 mM NaCl + MT) compared to the control. Car content in *A. corniculatum* was higher by 22.8% under T4 (400 mM NaCl). In *K. obovata*, Car content was 20% higher under T7 (400 mM NaCl + MT) than under the control. In the case of *R. stylosa*, the highest Car content was recorded in the control treatment, and Car content slightly decreased under all salinity and melatonin treatments. Total chlorophyll showed species- and salinity-dependent responses. In *A. corniculatum*, Chl-T was 17.6% and 5.5% higher under T5 and T6 than under the corresponding T2 and T3 treatments, respectively, but was 15.6% lower under T7 than T4. In *K. obovata*, Chl-T was 1.8%, 58.0%, and 33.0% higher under T5, T6, and T7 than under the corresponding NaCl-only treatments. In *R. stylosa*, the corresponding changes were −13.9%, −1.3%, and +4.8%, respectively. These patterns indicate that the clearest MT-associated increase in total chlorophyll occurred in *K. obovata*, particularly at 200 mM NaCl.

**Figure 6:**
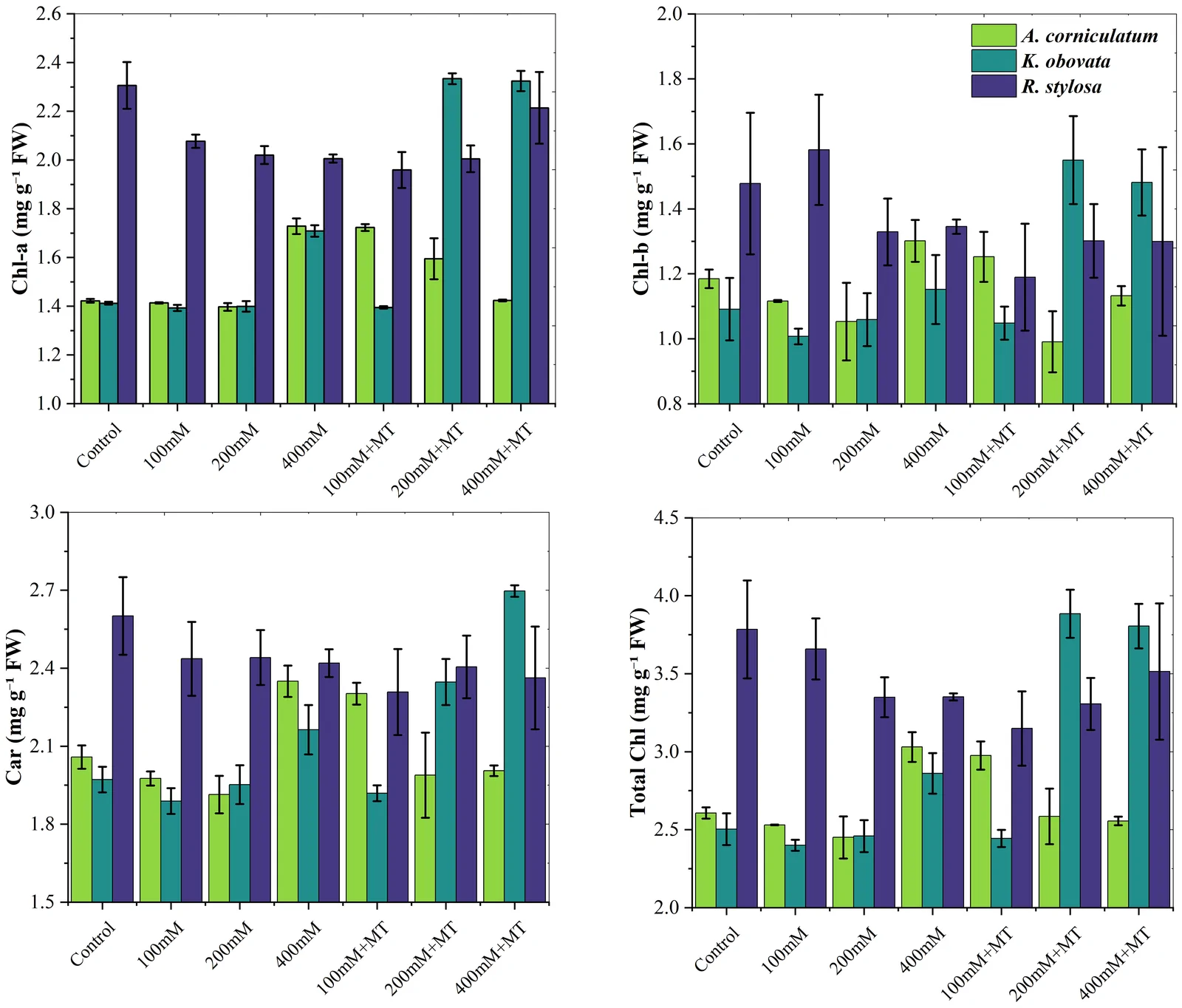
Effects of NaCl stress and melatonin application on photosynthetic pigment contents such as chlorophyll a (Chl–a), chlorophyll b (Chl–b), carotenoids (Car), and total chlorophyll of three mangrove species. Values are presented as mean ± SD of five seedling subsamples within each species × treatment unit. Error bars represent variability among seedling subsamples and do not represent independent treatment-level replication.

### 3.6 Comparative effects of melatonin across species and salinity levels

**Table 2** summarizes the descriptive responses of the three mangrove species to corresponding NaCl-only and NaCl + MT treatments. At 200 mM NaCl, *A. corniculatum* showed higher P_N_ (3.17 ± 0.98 to 7.74 ± 1.76), Gs (0.021 ± 0.007 to 0.097 ± 0.021), and Tr (0.00051 ± 0.00019 to 0.00186 ± 0.00123) under the corresponding NaCl + MT treatment, representing increases of 144.1%, 358.1%, and 266.4%, respectively. In *K. obovata*, total chlorophyll increased from 2.46 ± 0.10 to 3.88 ± 0.15 under 200 mM NaCl + MT, corresponding to a 58.0% increase. At the same salinity level, *R. stylosa* showed increases of 44.9%, 41.7%, and 28.4% in P_N_, Gs, and Tr, respectively. Overall, the magnitude and direction of MT-associated responses varied among species and salinity levels.

**Table 2:** Descriptive comparison of physiological and photosynthetic responses between NaCl-only and corresponding NaCl + melatonin treatments in three mangrove species.

Species	NaCl (mM)	P_N_	Gs	Tr	Fv/Fm	ΦPSII	NPQ	Chl-T
*A. corniculatum*	100	5.11 ± 0.89 → 6.70 ± 0.86 (+31.2%)	0.070 ± 0.031 → 0.098 ± 0.076 (+41.1%)	0.00141 ± 0.00006 → 0.00141 ± 0.00092 (-0.4%)	0.850 ± 0.006 → 0.851 ± 0.003 (+0.2%)	0.334 ± 0.013 → 0.338 ± 0.006 (+1.4%)	0.100 ± 0.009 → 0.397 ± 0.262 (+296.9%)	2.53 ± 0.00 → 2.98 ± 0.09 (+17.6%)
	200	3.17 ± 0.98 → 7.74 ± 1.76 (+144.1%)	0.021 ± 0.007 → 0.097 ± 0.021 (+358.1%)	0.00051 ± 0.00019 → 0.00186 ± 0.00123 (+266.4%)	0.809 ± 0.033 → 0.854 ± 0.008 (+5.6%)	0.410 ± 0.066 → 0.328 ± 0.023 (-20.1%)	0.177 ± 0.025 → 0.273 ± 0.154 (+53.8%)	2.45 ± 0.13 → 2.59 ± 0.18 (+5.5%)
	400	3.02 ± 2.07 → 6.15 ± 3.56 (+103.4%)	0.047 ± 0.045 → 0.047 ± 0.035 (+0.1%)	0.00069 ± 0.00039 → 0.00079 ± 0.00048 (+15.2%)	0.844 ± 0.008 → 0.841 ± 0.009 (-0.3%)	0.328 ± 0.020 → 0.345 ± 0.015 (+5.1%)	0.133 ± 0.096 → 0.504 ± 0.357 (+280.1%)	3.03 ± 0.09 → 2.56 ± 0.03 (-15.6%)
*K. obovata*	100	7.51 ± 3.73 → 7.67 ± 1.65 (+2.1%)	0.071 ± 0.012 → 0.037 ± 0.025 (-47.6%)	0.00153 ± 0.00031 → 0.00052 ± 0.00020 (-65.9%)	0.857 ± 0.014 → 0.835 ± 0.012 (-2.5%)	0.317 ± 0.029 → 0.351 ± 0.011 (+10.6%)	0.343 ± 0.096 → 0.347 ± 0.039 (+1.1%)	2.40 ± 0.04 → 2.44 ± 0.06 (+1.8%)
	200	6.24 ± 2.36 → 6.55 ± 3.15 (+5.0%)	0.052 ± 0.031 → 0.043 ± 0.018 (-18.5%)	0.00107 ± 0.00069 → 0.00079 ± 0.00036 (-26.7%)	0.839 ± 0.005 → 0.847 ± 0.015 (+1.1%)	0.350 ± 0.014 → 0.341 ± 0.022 (-2.6%)	0.430 ± 0.248 → 0.246 ± 0.047 (-42.8%)	2.46 ± 0.10 → 3.88 ± 0.15 (+58.0%)
	400	3.64 ± 1.72 → 5.22 ± 0.76 (+43.5%)	0.022 ± 0.021 → 0.018 ± 0.006 (-19.0%)	0.00033 ± 0.00019 → 0.00034 ± 0.00011 (+2.1%)	0.856 ± 0.005 → 0.848 ± 0.006 (-0.9%)	0.322 ± 0.012 → 0.337 ± 0.010 (+4.6%)	0.265 ± 0.204 → 0.430 ± 0.040 (+62.3%)	2.86 ± 0.13 → 3.81 ± 0.14 (+33.0%)
*R. stylosa*	100	16.56 ± 2.11 → 13.63 ± 7.43 (-17.7%)	0.139 ± 0.071 → 0.082 ± 0.092 (-41.1%)	0.00270 ± 0.00088 → 0.00114 ± 0.00122 (-57.8%)	0.841 ± 0.009 → 0.842 ± 0.003 (+0.2%)	0.340 ± 0.012 → 0.343 ± 0.002 (+1.0%)	0.638 ± 0.370 → 0.300 ± 0.202 (-53.0%)	3.66 ± 0.20 → 3.15 ± 0.24 (-13.9%)
	200	11.26 ± 5.48 → 16.32 ± 3.02 (+44.9%)	0.072 ± 0.022 → 0.102 ± 0.067 (+41.7%)	0.00159 ± 0.00051 → 0.00204 ± 0.00191 (+28.4%)	0.857 ± 0.006 → 0.852 ± 0.009 (-0.7%)	0.335 ± 0.016 → 0.353 ± 0.016 (+5.3%)	0.457 ± 0.270 → 0.352 ± 0.273 (-23.1%)	3.35 ± 0.13 → 3.31 ± 0.17 (-1.3%)
	400	13.81 ± 0.27 → 11.82 ± 5.09 (-14.4%)	0.058 ± 0.021 → 0.058 ± 0.045 (-0.4%)	0.00117 ± 0.00017 → 0.00091 ± 0.00058 (-22.2%)	0.831 ± 0.023 → 0.839 ± 0.002 (+0.9%)	0.366 ± 0.038 → 0.346 ± 0.005 (-5.4%)	0.360 ± 0.047 → 0.317 ± 0.045 (-11.9%)	3.35 ± 0.02 → 3.51 ± 0.44 (+4.8%)

Each parameter is shown as NaCl-only mean ± SD → corresponding NaCl + MT mean ± SD (percentage change). Values represent five seedling subsamples within each species × treatment unit. Percentage changes were calculated from unrounded mean values; displayed means ± SD are rounded for presentation.

## 4 Discussion

The aim of the current study was to compare physiological and growth responses of mangrove seedlings between NaCl-only and corresponding NaCl + MT treatments. Previous studies have reported that MT can enhance plant salinity tolerance through stress-responsive pathways, antioxidant defense, and reduced oxidative damage (Ali et al., 2024; Ashraf et al., 2026). These mechanisms were not directly measured in the present study. Therefore, the improved performance of melatonin-treated seedlings under NaCl stress would suggest that exogenous melatonin helps mangrove seedlings maintain better physiological activities and growth under saline conditions.

Leaf area expansion provides an integrated indicator of seedling growth under salinity stress because it reflects photosynthetically active surface development and the capacity to maintain growth under stress. The logistic growth models adequately described leaf-area development across species and treatments, with R^2^ values ranging from 0.962 to 0.998. These high R^2^ values indicate good agreement between the model and observed growth trajectories, although model fit itself should not be interpreted as evidence of treatment effectiveness. However, the response varied among species and salinity levels. At 200 mM NaCl, K was 14.6% and 43.0% higher under NaCl + MT than under NaCl alone in *A. corniculatum* and *K. obovata*, respectively. Previous studies have reported that salinity stress suppresses leaf growth by reducing water uptake, cell enlargement, and photosynthetic capacity, whereas exogenous melatonin can enhance plant tolerance by regulating oxidative stress and stress-responsive physiological pathways (Khan et al., 2024).

Gas-exchange responses further demonstrated a strong species- and salinity-dependent pattern in the MT-associated responses. In *A. corniculatum*, the clearest improvement occurred at 200 mM NaCl + MT, where the reductions in P_N_ and Tr were substantially smaller than under 200 mM NaCl alone. This coordinated response of P_N_, G_S_, and Tr is consistent with partial maintenance of stomatal gas exchange and CO_2_ assimilation under moderate salinity. In *K. obovata*, P_N_ was higher under some NaCl + MT treatments than under the corresponding NaCl-only treatments, although photosynthetic performance remained constrained as salinity increased. In contrast, *R. stylosa* maintained comparatively greater gas-exchange performance under NaCl stress itself and showed a smaller relative dependence on MT, suggesting stronger inherent physiological tolerance to salinity. Similar findings have been reported in salt-stressed plants, where exogenous melatonin improved photosynthetic capacity by regulating stomatal conductance, maintaining chlorophyll fluorescence, and strengthening antioxidant defense (Jiang et al., 2021; Dou et al., 2025). In mangroves, salinity-induced reductions in photosynthesis are commonly linked to stomatal limitation and restricted water exchange, particularly in less tolerant species (Chen et al., 2022). A research study reported that activation of endogenous melatonin biosynthesis helped *K. obovata* seedlings maintain photosynthetic capacity and PSII efficiency under stress, indicating that melatonin-related pathways are important for mangrove stress resilience (Shan et al., 2025). Furthermore, salt-induced reductions in photosynthesis and stomatal conductance have been reported in mangroves and other plants, where salinity restricts CO_2_ diffusion, water loss, and photosynthetic efficiency (Solangi et al., 2021).

The higher P_N_, Gs, and Tr values observed in *A. corniculatum* under 200 mM NaCl + MT compared with 200 mM NaCl alone were consistent with partial maintenance of stomatal gas exchange under moderate salinity. In salt-stressed mango, MT application increased antioxidant enzyme activity and improved ion balance by increasing K and Ca while reducing Na and Cl, supporting possible roles of antioxidant regulation and ion homeostasis in MT-mediated salinity tolerance (Hassan et al., 2025). However, the comparatively small MT-associated changes in *K. obovata* indicate that photosynthetic limitations remained under increasing salinity, particularly when stomatal conductance was low. In *R. stylosa*, the smaller relative response to MT suggests that its physiological response to salinity differed from those observed in the other species. Chlorophyll-fluorescence responses showed that maximal PSII efficiency (Fv/Fm) remained comparatively stable across most NaCl and NaCl + MT treatments, indicating that the treatments caused relatively limited changes in maximum PSII photochemical efficiency. Therefore, the small treatment differences in Fv/Fm should not be interpreted as evidence of a major restoration of PSII reaction center function by MT. Instead, treatment-related adjustments were more apparent in NPQ and, in some cases, ΦPSII. In *A. corniculatum*, NPQ increased strongly in the NaCl + MT treatments, consistent with enhanced engagement of non-photochemical energy dissipation under excess excitation pressure.

A recent study focused on cold stress reported MT-associated protection of photosynthetic electron transport and regulation of redox-related pathways in mangroves (Li et al., 2024). These literature-reported mechanisms may also be relevant under salt stress, where oxidative damage, photoinhibition, and reduced carbon assimilation can contribute to growth inhibition; however, these mechanisms were not directly assessed in the present study (Li et al., 2026). In *K. obovata*, NPQ values were particularly high under 200 mM NaCl (T3) and 400 mM NaCl + MT (T7), indicating strong engagement of non-photochemical energy dissipation under salt stress. In *R. stylosa*, ΦPSII increased in moderate and high salinity. Previous research has shown that ΦPSII more sensitive indicators of photochemical adjustment and photoprotection (Li et al., 2017). Another study showed that MT can improve chlorophyll fluorescence performance under salinity by protecting PSII, reducing oxidative damage, and limiting chlorophyll degradation (Moradi et al., 2026). Overall, *R. stylosa* showed comparatively stable PSII performance, *K. obovata* showed relatively high NPQ under some treatments, and *A. corniculatum* showed the clearest MT-associated increase in NPQ under salinity. In this study photosynthetic pigment responses also varied among species and salinity levels following MT supplementation. Previous studies have shown that salinity reduces photosynthetic pigments through impaired biosynthesis, pigment degradation, and oxidative damage, whereas melatonin helps preserve them by enhancing antioxidant protection and chloroplast stability (Karumannil et al., 2023; Wang et al., 2024). Similar melatonin-induced improvement in chlorophyll and carotenoid accumulation has also been reported in salt-stressed crops, supporting its role in maintaining photosynthetic pigment systems under adverse conditions (Askari et al., 2023). Overall, *K. obovata* showed the largest MT-associated increase in total chlorophyll under moderate salinity, whereas *R. stylosa* and *A. corniculatum* showed smaller and more variable pigment responses to MT supplementation.

Overall, the observed responses were strongly species- and salinity-dependent. *A. corniculatum* showed the clearest MT-associated increase in gas-exchange performance under moderate salinity, *K. obovata* showed prominent changes in pigment content and NPQ, whereas *R. stylosa* maintained comparatively stable physiological and PSII performance under salinity even without MT supplementation. These patterns indicate that MT supplementation was associated with changes in several measured physiological traits; however, the underlying antioxidant, ion-regulatory, chloroplast, and molecular signaling mechanisms were not directly measured and therefore cannot be established from the present experiment. Further independently replicated and field-based studies are required to evaluate the practical significance of these responses.

This study has three main design limitations. First, a melatonin only treatment was not included because the primary objective was to compare seedling responses under NaCl stress with and without exogenous melatonin, rather than to evaluate the independent effect of melatonin under non-saline conditions. Therefore, the present design specifically focuses on the comparative role of melatonin in modifying salinity responses. Second, the five seedlings within each species × treatment combination shared one container reservoir unit and therefore represented biological subsamples rather than independent treatment replicates. Consequently, each species × treatment combination contained only one independent experimental treatment unit, and treatment responses were interpreted descriptively rather than through conventional inferential comparisons. Third, ethanol was used as the solvent for MT in T5–T7, whereas the corresponding non-MT treatments did not receive an ethanol carrier control. Therefore, a potential minor effect of the ethanol carrier cannot be completely excluded.

## 5 Conclusion

The current study assessed growth, photosynthetic, chlorophyll-fluorescence, gas-exchange, and pigment responses under NaCl-only and corresponding NaCl + MT treatments in *A. corniculatum*, *K. obovata*, and *R. stylosa*. Growth-model estimates showed species-specific patterns; at 200 mM NaCl, K was higher under the corresponding NaCl + MT treatment than under NaCl alone in *A. corniculatum* and *K. obovata*. The logistic growth model provided a good descriptive fit to the observed leaf-area trajectories (R^2^ = 0.962–0.998). At 400 mM NaCl (T4), P_N_ was lower in *A. corniculatum* and *K.* obovata. Among the three species, *R. stylosa* generally maintained comparatively higher P_N_ and G_S_ across most treatments.

Chlorophyll fluorescence parameters showed relatively stable Fv/Fm values across treatments, indicating limited changes in maximum PSII photochemical efficiency. Treatment-related changes were more apparent in ΦPSII and NPQ and varied among species and salinity levels. At 200 mM NaCl, *A. corniculatum* showed higher gas-exchange values under NaCl + MT than under NaCl alone, while *K. obovata* showed higher leaf-growth potential and total chlorophyll under the corresponding NaCl + MT treatment. Overall, MT supplementation was associated with species- and salinity-dependent changes in growth, gas exchange, chlorophyll fluorescence, and photosynthetic pigments. The present study did not measure molecular, antioxidant, or ion-regulatory mechanisms; therefore, these mechanisms cannot be established from the current results. The observed physiological responses suggest that MT may have potential relevance for mangrove nursery cultivation and restoration applications under increasingly saline coastal conditions. However, independently replicated experiments and field validation are required before practical nursery or restoration recommendations can be made.

## Data Availability

The raw data supporting the conclusions of this article will be made available by the authors, without undue reservation.
